# Comparative proteomic assessment of matrisome enrichment methodologies

**DOI:** 10.1042/BCJ20160686

**Published:** 2016-10-27

**Authors:** Lukas Krasny, Angela Paul, Patty Wai, Beatrice A. Howard, Rachael C. Natrajan, Paul H. Huang

**Affiliations:** 1 Division of Cancer Biology, The Institute of Cancer Research, 237 Fulham Road, London SW3 6JB, U.K.; 2 Proteomics Core Facility, The Institute of Cancer Research, London SW3 6JB, U.K.; 3 The Breast Cancer Now Toby Robins Research Centre, Division of Breast Cancer Research, The Institute of Cancer Research, London SW3 6JB, U.K.; 4 Division of Molecular Pathology, The Institute of Cancer Research, London SW3 6JB, U.K.

**Keywords:** extracellular matrix, mass spectrometry, matrisome, proteomics, tissue extraction

## Abstract

The matrisome is a complex and heterogeneous collection of extracellular matrix (ECM) and ECM-associated proteins that play important roles in tissue development and homeostasis. While several strategies for matrisome enrichment have been developed, it is currently unknown how the performance of these different methodologies compares in the proteomic identification of matrisome components across multiple tissue types. In the present study, we perform a comparative proteomic assessment of two widely used decellularisation protocols and two extraction methods to characterise the matrisome in four murine organs (heart, mammary gland, lung and liver). We undertook a systematic evaluation of the performance of the individual methods on protein yield, matrisome enrichment capability and the ability to isolate core matrisome and matrisome-associated components. Our data find that sodium dodecyl sulphate (SDS) decellularisation leads to the highest matrisome enrichment efficiency, while the extraction protocol that comprises chemical and trypsin digestion of the ECM fraction consistently identifies the highest number of matrisomal proteins across all types of tissue examined. Matrisome enrichment had a clear benefit over non-enriched tissue for the comprehensive identification of matrisomal components in murine liver and heart. Strikingly, we find that all four matrisome enrichment methods led to significant losses in the soluble matrisome-associated proteins across all organs. Our findings highlight the multiple factors (including tissue type, matrisome class of interest and desired enrichment purity) that influence the choice of enrichment methodology, and we anticipate that these data will serve as a useful guide for the design of future proteomic studies of the matrisome.

## Introduction

The extracellular matrix (ECM) in the tissue microenvironment is a complex and heterogeneous collection of proteins that play important roles in tissue development and homeostasis [1]. In addition to functioning as scaffolds that confer structural integrity in mammalian tissues, ECM components provide biochemical and biophysical cues which are transmitted by cell surface receptors, for instance the integrins, to trigger intracellular signalling events that regulate fundamental cell decisions including proliferation, death and differentiation [2,3]. It is therefore unsurprising that dysregulation in ECM biology is associated with many pathologies such as cancer, fibrosis, atherosclerosis, arthritis and a range of genetic disorders [3–8]. To provide a consensus for the classification of this class of proteins, there has been a recent effort to develop an *in silico* definition of the ECM and its associated proteins, collectively designated as the ‘matrisome’ [9]. The matrisome is composed of the ‘core matrisome’, which consists of ECM glycoproteins, collagens and proteoglycans and the ‘matrisome-associated proteins’ that include ECM regulators, affiliated proteins and secreted factors [10]. The early draft of the matrisome was intended to be inclusive, and the subcategories within matrisome-associated proteins as designated by Naba et al. [9] contain multiple proteins that are predicted to interact with the core matrisome. It should therefore be noted that the definition of these subcategories remains loose and the interaction of many of these matrisome-associated components with the ECM remains to be experimentally confirmed.

While the matrisome provides an *in silico* framework for classifying ECMs and their associated proteins, the exact composition of the ECM components *in vivo* varies between tissues and organisms [10]. Comprehensive experimental characterisation of the matrisome using proteomics has been challenging in part because many ECM proteins are large, highly glycosylated proteins that are frequently insoluble due to the presence of covalent cross-links [11]. Furthermore, the matrisome is composed of a large number of proteins with a wide dynamic range, necessitating the use of enrichment strategies [11]. Several approaches have been developed to enrich for core matrisome components and preserve matrisome-associated proteins while removing contaminating intracellular proteins. These matrisome enrichment strategies are broadly split into methods that employ decellularisation of intact tissue and those that extract ECM from native or crude tissue homogenate [11].

Decellularisation involves the use of weak detergents, trypsinization or low ionic strength buffers to disrupt cell membranes leading to the release of intracellular proteins into solution while preserving the insoluble ECM scaffold [12]. This approach is commonly used in the generation of scaffolds for tissue engineering and has been applied to different tissue types, including rat lung and human and porcine myocardium [13–15]. Methods that are routinely employed to extract ECM from native tissue homogenate include the use of differential detergent extractions to sequentially fractionate distinct cellular compartments (cytosolic, nuclear, membrane and cytoskeletal), leaving an insoluble fraction that contains the ECM [9]. This approach has been deployed to characterise the matrisome in murine lung and mammary gland as well as human colon and liver [9,16–18]. Another strategy for extraction of ECM from native tissue homogenate involves the use of high salt buffers to remove intracellular components followed by solubilisation of the remaining pellet in a chaotrope (usually urea). This method has been successfully utilised in multiple studies to catalogue ECM proteins in rat mammary gland [19,20]. In an elaboration of this method, Hill et al. [13] performed a further chemical digestion step to aid in the solubilisation of the urea-insoluble fraction prior to trypsin digestion, improving the recovery of matrisome components identified in rat lungs. Treatment by alkaline detergent has been used to enrich matrisomal components from murine glomeruli [21,22]. The alkaline detergent improves the solubilisation of cellular components and disrupts cell–ECM interactions. Additionally, a combination of sequential extraction and decellularisation techniques has successfully been applied to enrich for cardiac ECM [23]. In this protocol, homogenised samples were first treated with high salt buffer and the resulting pellet was subjected to decellularisation in SDS solution. Decellularised samples were then homogenised in guanidium-HCl buffer prior to proteomic analysis.

Given the diverse strategies available for matrisome enrichment, it is currently unknown how the performance of the different methodologies compares in the proteomic identification of matrisome components across multiple tissue types. This knowledge will be necessary for the development of the proposed ‘ECM atlas’ that seeks to compile the ECM composition of different tissues as defined by mass spectrometry [10]. In this study, we perform a comparative proteomic assessment of four matrisome enrichment methodologies (two decellularisation and two native extraction approaches) in four murine organs (heart, mammary gland, lung and liver). We sought to address two important questions. (1) Do different enrichment methodologies introduce bias or sample loss in the identification of core matrisome and matrisome-associated proteins within the same organ? (2) Does tissue type influence the recovery of matrisome components across the different extraction methods? Our data find that while SDS decellularisation leads to high matrisome enrichment efficiency across all studied tissue types, the chemical digestion method by Hill et al. [13] consistently identified more matrisome proteins in the four organs examined. Moreover, we show a clear benefit of matrisome enrichment over unenriched tissue for the identification of ECM components in murine liver and heart. We find that Triton X-100 decellularisation is superior in enriching for proteoglycan components of the core matrisome compared with other enrichment methods. Our data also point to significant losses in matrisome-associated proteins across all matrisome enrichment methodologies evaluated in the present study compared with non-enriched samples. Our results demonstrate that the choice of matrisome enrichment methodology is a key factor in determining both the enrichment efficiency and the number and type of matrisome proteins identified *in vivo*.

## Experimental section

### Animal models and tissue collection

All animal work was carried out under UK Home Office project and personal licences following local ethical approval from the Institute of Cancer Research Ethics Committee and in accordance with local and national guidelines. Hearts, livers, lungs and the number 3 mammary glands were dissected from 10- to 14-week-old virgin female severe combined immunodeficiency disease Beige mice. Tissues were snap-frozen in liquid nitrogen directly after the surgery and stored at −80°C.

### Tissue processing and ECM protein enrichment

Tissues were cut into small pieces, weighed and placed into precooled tubes for decellularisation or sequential extraction. Three biological and two technical replicates were performed for each matrisome enrichment method.

Triton X-100 decellularisation (based on Xu et al. [12]): samples (10–100 mg) were placed into polybuffered saline (PBS) solution with 10 Kallikrein inhibitor unit (KIU)/ml aprotinin (Sigma) and washed for 48 h at 4°C. Samples were then decellularised with a solution of Tris–HCl (10 mM, pH 8), 3% Triton X-100 (Sigma), 0.1% ethylenediamine tetraacetic acid (EDTA, Sigma) and 10 KIU/ml aprotinin for 72 h at 4°C. In the next step, samples were incubated in a solution of 0.2 µg/ml ribonuclease A (Sigma) and 0.2 mg/ml deoxyribonuclease I (Sigma) in 50 mM Tris–HCl (pH 7.4), 10 mM MgCl_2_ (Sigma) and 5 mM CaCl_2_ (Sigma) for 24 h at 37°C. After 24 h of washing in PBS at 4°C, decellularised samples were homogenised in 250 µl of 8 M urea (Sigma), 100 mM ammonium bicarbonate (ABC, Sigma) using the LabGEN700 homogeniser (Cole-Parmer, UK); protein concentration was measured by a bicinchoninic acid (BCA) assay (Thermo Scientific) and homogenates were frozen and stored at −80°C until protein digestion. During all washing and decellularisation steps, samples were slowly rotated and solutions were refreshed every 24 h.

SDS decellularisation (based on Xu et al. [12]): samples (10–100 µg) were placed into PBS solution with 10 KIU/ml aprotinin and washed for 48 h at 4°C. Then, samples were decellularised with a solution of Tris–HCl (10 mM, pH 8), 0.5% SDS (Sigma), 0.1% EDTA and 10 KIU/ml aprotinin for 72 h at 4°C. Subsequent steps are the same as for decellularisation by Triton X-100. During all washing and decellularisation steps, samples were slowly rotated and solutions were refreshed every 24 h.

Extraction method A (EMA; based on Hill et al. [13]): samples were homogenised in 0.5 ml of Tris–HCl (50 mM, pH 7.4), 0.25% 3-[(3-cholamidopropyl)dimethylammonio]-1-propanesulfonate hydrate (CHAPS, Sigma), 25 mM EDTA, 3 M NaCl (Sigma) and 10 KIU/ml aprotinin by the LabGEN700 homogeniser. Homogenised tissue was spun at 15 000 rpm, 4°C and the supernatant was removed. The pellet was resuspended in 0.5 ml of the same homogenisation buffer and additionally washed two times. The pellet was then washed by 0.3 ml of 8 M urea, 100 mM ABC and 25 mM tris(2-carboxyethyl)phosphine (Sigma) and spun. The resulting pellet was resuspended in 86% trifluoroacetic acid (TFA; Sigma) and 100 mM CNBr (Sigma), and incubated at 25°C overnight in the dark. After digestion, samples were washed three times by H_2_O and dried by SpeedVac (Thermo Scientific, UK). After resuspending in a solution of 8 M urea and 100 mM ABC, protein concentration was measured by the BCA assay and samples were stored at −80°C until digestion by trypsin.

Extraction method B (EMB; based on by Naba et al. [9]): samples were extracted using the Compartmental Extraction Kit (Millipore) as per the manufacturer's instructions. Briefly, tissues were homogenised in cold buffer C using the LabGEN700 homogeniser, rotated at 4°C for 20 min and spun at 15 000 rpm, 4°C for 20 min. The pellet was then washed by cold buffer W, rotated for 5 min at 4°C and spun. Nuclear proteins were removed by resuspending the pellet in cold buffer N. After rotating for 20 min at 4°C and centrifugation, the supernatant was removed and the pellet was resuspended in buffer M, rotated for 20 min at 4°C and spun. CS buffer pre-warmed to room temperature was added to the pellet, rotated for 20 min at room temperature and spun. The pellet was immediately washed, resuspended in cold buffer C, rotated for 5 min at 4°C and spun. The final pellet was resuspended in solution of 8 M urea and 100 mM ABC and protein concentration was measured by BCA assay and stored at −80°C. Protease inhibitors were added to all extraction buffers.

Non-enriched sample: tissue was homogenised in solution of 8 M urea, 50 mM ABC and 75 mM NaCl with protease inhibitors by the LabGEN700 homogeniser. Proteins were directly precipitated by 1.6 ml of ice cold acetone (VWR Chemicals) overnight at −20°C. The precipitate was centrifuged at 15 000 rpm, 4°C for 20 min and resuspended in solution of 8 M urea and 100 mM ABC. The protein concentration was measured by the BCA assay and samples were stored at −80°C.

### Protein digestion and sample preparation

A total of 20 µg of protein for each sample were reduced by 20 mM dithiothreitol (Sigma) at 56°C for 40 min and alkylated by 30 mM iodoacetamide (Sigma) at 25°C for 25 min in the dark. After dilution of the protein sample to final concentration of 2 M urea and 100 mM ABC, all samples except those extracted by the EMA protocol were deglycosylated by 0.2 µg of protein-*N*-glycosidase F (New England Biolabs). Samples extracted by the EMB protocol were digested by 0.2 µg of Endoproteinase Lys-C (Sigma) at 37°C for 2 h and subsequently by 0.4 µg of trypsin (Life Technologies Ltd) at 37°C overnight. Other samples were directly digested by 0.4 µg of trypsin at 37°C overnight. Digestion was stopped by acidification of the solution using 10% TFA (Sigma).

After digestion, samples were desalted by solid phase extraction using C18 OMIX tips (Agilent) or OPTI trap (Optimize Technologies), dried by SpeedVac and dissolved in 2% acetonitrile (ACN, VWR Chemicals)/0.1% formic acid (FA, Sigma). Concentration of peptides after digestion and desalting was measured by the BCA assay.

### Mass spectrometry

Samples were analysed by liquid chromatography–tandem mass spectrometry (LC–MS/MS) using the LTQ Orbitrap Velos (Thermo Scientific) mass spectrometer coupled online with the Eksigent nanoLC automated system. A sample (1 µg of total digest) was loaded onto the Thermo Acclaim PepMap 100 C18 (100 µm × 2 cm, 5 µm) guard column and separated on an in-house packed column (75 µm × 28 cm, Reprosil-Pur C18-AQ 3 µm particles) using the linear gradient from 5 to 40% ACN/0.1% FA in 88 or 240 min for analysis of non-enriched samples with a long gradient. The constant flow rate of 300 nl/min was used for all samples.

Mass spectra were acquired in the data-dependent mode using the Thermo Xcalibur (ver. 2.2.42) software in the mass range from *m*/*z* 375 to 2000. The 20 most intense precursor ions were selected for fragmentation by collision-induced dissociation with normalised collision energy 40 and dynamic exclusion for 40 s within a mass range of ±10 ppm and with a repeat count of 1.

### Data processing and analysis

MS/MS mass spectra of precursors with charge states of 2^+^, 3^+^ and 4^+^ were extracted by Proteome Discoverer (ver. 1.4, Thermo Scientific). All MS/MS spectra were analysed using an in-house Mascot server (ver. 2.3.02, Matrix Science), which searched the Uniprot database selected for *Mus musculus* (16 769 entries, downloaded on 29 April 2015), with the enzyme specificity set as C-terminal lysine and arginine or CNBr + C-terminal lysine and arginine and two possible missed cleavages. Mascot was searched with a fragment ion mass tolerance of 0.6 Da and a parent ion tolerance of 10 ppm. Carbamidomethylation of cysteine was specified as a fixed modification, while oxidation of lysine, methionine and proline and acetylation of lysine and the N-terminus were specified as variable modifications.

Scaffold (ver. 4.4.6, Proteome Software, Inc.) was used to validate MS/MS-based peptide and protein identifications. Peptide identifications were accepted if they could achieve a false discovery rate (FDR) of <1.0% by the Scaffold Local FDR algorithm. Protein identifications were accepted if they could be established at >95.0% probability and contained at least two identified peptides. Protein probabilities were assigned by the Protein Prophet algorithm [24]. Proteins that contained similar peptides and could not be differentiated based on MS/MS analysis alone were grouped to satisfy the principles of parsimony.

Proteins identified by the Mascot search and validated by Scaffold were then annotated using the mouse matrisome database MatrisomeDB from http://matrisomeproject.mit.edu/ (downloaded on 21 October 2015).

### Statistical analysis

‘Percentage protein extraction yield’ is defined as (amount of protein extracted from tissue) × 100/(wet weight of the tissue). Statistical evaluation of percentage extraction yields across the different matrisome enrichment methods was performed by analysis of variance with the Tukey-corrected multiple comparisons, and plots were generated using the GraphPad Prism6 software (ver. 6.07). The value of *P* < 0.05 was considered statistically significant.

Datasets from both technical replicates were combined and only proteins identified in at least two of three biological replicates were considered. Proteins annotated using the MatrisomeDB were assigned as matrisomal and the ‘percentage matrisome enrichment’ is defined as (number of matrisomal proteins) × 100/(total number of proteins identified). The online tool Venny 2.1 (http://bioinfogp.cnb.csic.es/tools/venny/index.html) was used for comparison of identified matrisomal proteins and for generation of Venn diagrams.

## Results and discussion

The aim of the present study was to evaluate four published protocols for matrisome enrichment in terms of (1) protein extraction yield, (2) the effectiveness of matrisome enrichment and (3) the number of unique matrisomal proteins identified by LC–MS/MS analysis. We utilised two decellularisation methods and two extraction methods to characterise four murine organs (heart, mammary gland, lung and liver). Identified matrisomal proteins were compared across the four methods to determine the relative ability of each method to enrich for matrisomal components. All enrichment experiments were performed in three biological replicates with two technical repeats.

### Matrisome enrichment workflows

**Figure 1.**
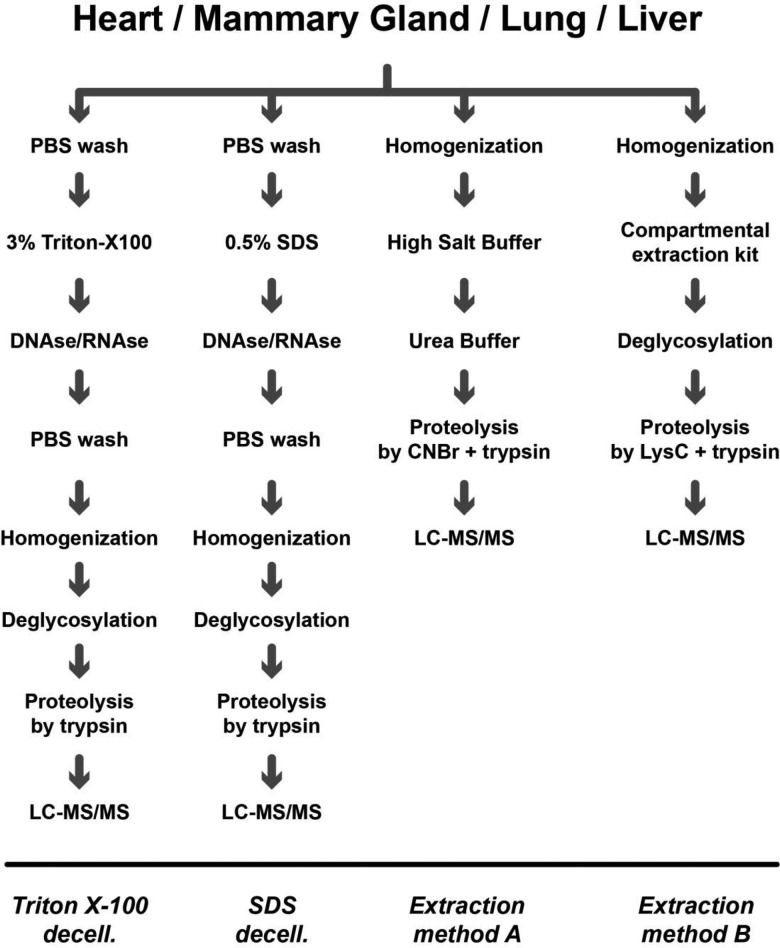
Matrisome enrichment workflows Schematic of the workflow depicting key sample processing steps during matrisome enrichment by four individual methods and subsequent LC–MS/MS analysis. LysC, endoproteinase LysC.

Decellularisation by anionic (SDS) or non-ionic (Triton X-100) detergents is the most frequently used approach in tissue engineering for the preparation of collagenous scaffolds [25]. After immersing the tissue in buffer with detergent to disrupt and remove cellular components, the decellularised scaffolds can then be homogenised in 8 M urea and digested by proteases for proteomic analysis of matrisomal components (Figure 1). However, decellularisation techniques were originally developed for the purposes of tissue engineering where the complete removal of cellular proteins is required to avoid potential undesirable immune responses [26]. Such complete decellularisation requires at least several hours to days of extensive physical and chemical treatment which may lead to protein degradation.

In contrast with decellularisation, two recently developed protocols for the extraction of matrisomal proteins from homogenised tissue offer a more rapid approach [11] (Figure 1). The principle is based on sequential removal of soluble cellular components after tissue homogenisation with the remaining insoluble fraction containing enriched matrisomal components. In the present study, we have employed two extraction methods: EMA is based on the protocol by Hill et al. [13] and EMB by Naba et al. [9]. EMA employs a buffer containing CHAPS and high ionic strength (3 M NaCl) to lyse cells and extract soluble cellular proteins while minimising matrisomal loss (Figure 1). Matrisomal proteins are then denatured and solubilised by 8 M urea, resulting in a fraction of soluble ECM and a pellet of insoluble ECM [13]. The insoluble pellet is then treated with CNBr to decrease the complexity of the protein meshwork and improve accessibility for trypsin prior to mass spectrometry analysis (Figure 1). EMB employs a commercial cellular compartment enrichment kit to sequentially remove cellular components (cytoplasmic, nuclear, membrane and cytoskeletal proteins) from tissue (Figure 1) [9]. The resultant pellet (which contains the matrisomal components) is solubilised in 8 M urea, deglycosylated and digested with LysC to decrease the complexity of the ECM meshwork prior to digestion with trypsin and mass spectrometry analysis (Figure 1).

In the present study, we applied a shotgun proteomic approach using nanoflow reversed-phase liquid chromatography coupled online to the Orbitrap Velos mass spectrometer. Of the 200–300 proteins which were identified in a typical sample after enrichment using this setup, 30–70 were assigned as matrisomal proteins. It should be noted that the number of identified proteins may vary on the LC–MS system used as well as the stringency employed during data processing (see Experimental section for details). No additional fractionation (SDS–PAGE and off-gel electrophoresis) was used in the present study.

### Heart

The heart is composed of myocardial muscle and collagenous heart valves and because of the periodic contractions of the myocardium, there are high demands for tissue flexibility and contractility [27]. The ECM in the heart is necessary for the distribution of mechanical forces throughout the myocardium and transmission of electromechanical signals maintaining systolic and diastolic function [28,29]. Dysregulation of ECM such as the increased accumulation of collagen is associated with the development of cardiovascular disease [29,30].

**Figure 2.**
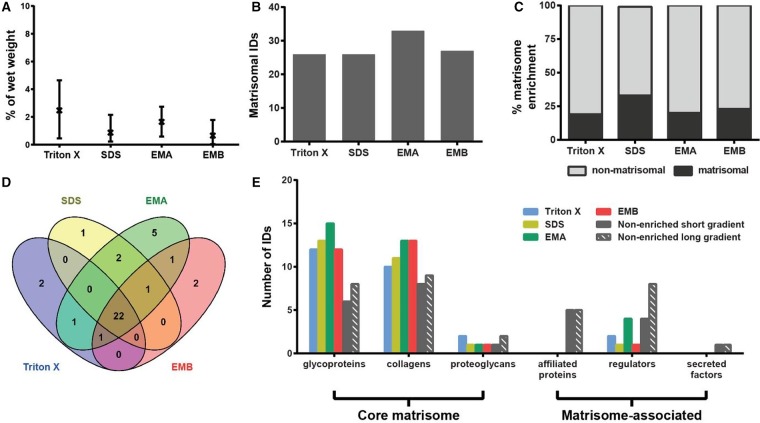
Comparison of methods for enrichment of the heart matrisome. (**A**) Percentage protein extraction yield after enrichment by individual methods. Results from three biological replicates are shown as mean and range (*n* = 3). (**B**) Number of matrisomal proteins identified (IDs) after enrichment. (**C**) Proportional representation of identified matrisomal and non-matrisomal proteins after enrichment. (**D**) Venn diagram depicting overlap of identified matrisomal proteins across the individual enrichment methods. (**E**) Distribution of proteins identified (IDs) in matrisomal classes for enriched and non-enriched samples. Only proteins detected in at least two biological replicates were considered.

Comparative assessment of the protein extraction yield (defined as the percentage of extracted protein as a function of organ wet weight) from heart shows an average yield of 0.66–2.47%, with no statistical difference across the four methods (Figure 2A). The EMA protocol identified the highest number of matrisomal proteins in the heart with 33 proteins found in at least two of three biological replicates (Figure 2B). However, when percentage matrisome enrichment (defined as the percentage of identified proteins which are catalogued in the Matrisome Project Database) is considered, decellularisation by SDS performed best, with 33% of total identified proteins being assigned as matrisome proteins (Figure 2C). As depicted in the Venn diagram in Figure 2D, 22 proteins were commonly identified by all enrichment techniques, showing good overlap in their enrichment capability, with only a few proteins uniquely enriched by just one of the methods.

The proteomic data were mapped onto the mouse matrisome database (MatrisomeDB, http://matrisomeproject.mit.edu/), where matrisomal proteins are divided into the ‘core matrisome’ (encompassing glycoproteins, collagens and proteoglycans) and the ‘matrisome-associated proteins’ (encompassing affiliated proteins, regulators and secreted factors) [9]. Glycoproteins and collagens were the most represented matrisomal classes in heart in our analysis (Figure 2E and Table 1). The proteins identified in these two classes have previously been documented in human and porcine hearts. For example, Didangelos et al. [15] identified the glycoproteins fibronectin (FINC) and fibulins (FBLN), the proteoglycan perlecan (PGBM) and multiple collagens in human aorta, whereas Barallobre-Barreiro et al. [31,32] isolated the laminin class of glycoproteins from porcine heart. The method of enrichment in these studies is based on a combination of extraction and decellularisation as the tissue was diced into smaller pieces (∼15–20 mg/piece) but not homogenised or minced. Interestingly, we did not observe much enrichment of matrisome-associated proteins; in particular, there were no matrisome-affiliated proteins and secreted factors found in our analysis. These data are consistent with a previous study by de Castro Bras et al. [23] who enriched and analysed matrisomal proteins from the mouse left ventricle, but were unable to identify any matrisome-associated proteins in the insoluble fraction. One potential reason for the lack of matrisome-associated proteins could be the result of multiple steps of solubilisation and washes to remove intracellular components in the four matrisome enrichment strategies during which soluble matrisome-associated proteins are likely to be washed away.

**Table 1:** List of identified matrisomal proteins

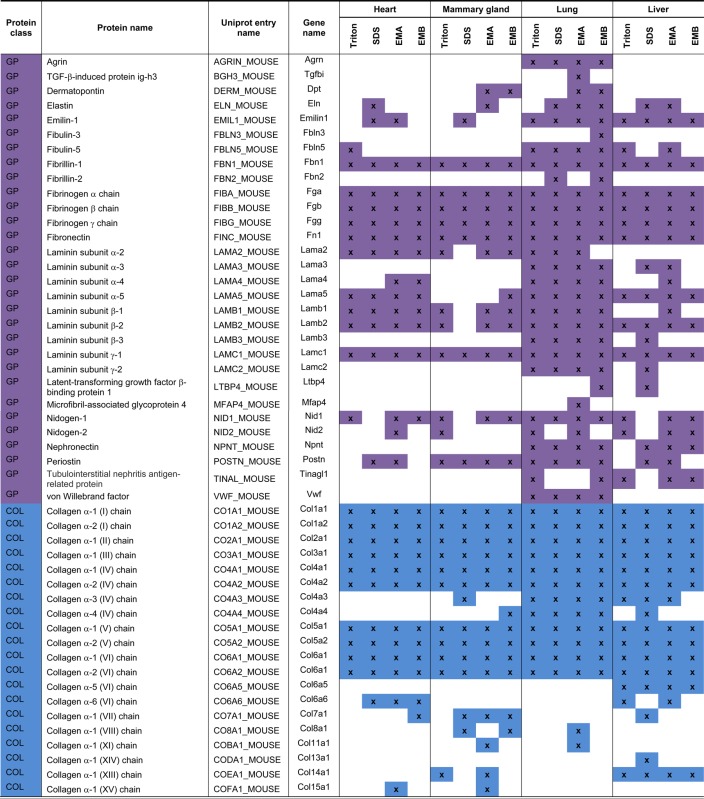
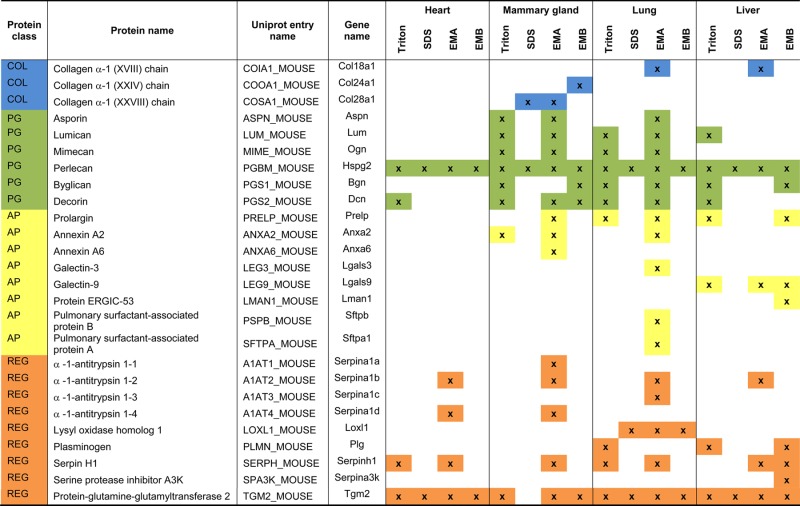

GP, glycoprotein; COL, collagen; PG, proteoglycan; AP, affiliated protein; REG, regulator.

To test this hypothesis, we performed an LC–MS/MS analysis of murine heart lysates that were not subjected to enrichment. While the non-enriched samples showed poorer recovery of glycoproteins and collagens, we identified 10 and 14 matrisome-associated proteins using a short (88 min) and long (4 h) gradient, respectively, in the non-enriched samples (Figure 2E). This finding demonstrates that matrisome enrichment leads to a significant loss of matrisome-associated proteins in the heart and that these components can be directly identified in non-enriched samples. Although the enrichment methodologies (in particular, the EMA protocol) were capable of enriching several ECM regulators, direct LC–MS/MS of non-enriched samples was superior with regard to the number of proteins identified in this class.

### Mammary gland

The mammary gland is composed of several lobules encapsulated and separated by basement membrane and connective tissue which is surrounded by a fatty layer that needs to be effectively removed before LC–MS/MS analysis [33]. Many matrisomal proteins have been reported as important players during mammary gland development and branching of ducts, including PGBM, matrix metalloproteinases and their inhibitors and transforming growth factor β (TGF-β) [33]. Moreover, the mammary gland stroma undergoes extensive changes and remodelling during pubertal development, pregnancy and after postpartum involution [34].

**Figure 3.**
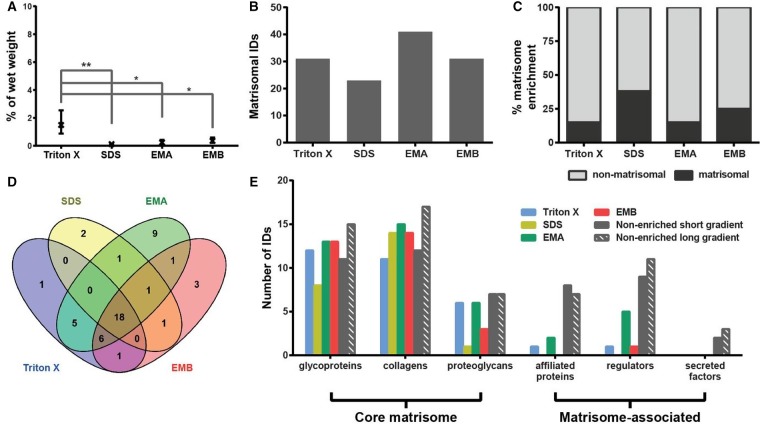
Comparison of methods for enrichment of the mammary gland matrisome. (**A**) Percentage protein extraction yield after enrichment by individual methods. Results from three biological replicates are shown as mean and range (*n* = 3). **P < 0.01, *P < 0.05. (**B**) Number of matrisomal proteins identified (IDs) after enrichment. (**C**) Proportional representation of identified matrisomal and non-matrisomal proteins after enrichment. (**D**) Venn diagram depicting the overlap of identified matrisomal proteins across the individual enrichment methods. (**E**) Distribution of proteins identified (IDs) in matrisomal classes for enriched and non-enriched samples. Only proteins detected in at least two biological replicates were considered.

Analysis of the protein extraction yields revealed that Triton X-100 decellularisation achieved the best recovery with an average of 1.5% wet weight (Figure 3A). The other three methods showed low extraction yield with SDS decellularisation yielding the lowest average of 0.11%. The highest number of 41 matrisomal proteins was identified in the samples extracted by the EMA protocol (Figure 3B). Conversely, SDS decellularisation resulted in the identification of the lowest number of 23 matrisomal proteins. Similar to murine heart, SDS decellularisation also showed the highest matrisomal enrichment in the mammary gland of 38% (Figure 3C). A total of 18 matrisome proteins were commonly identified by all methods, whereas 9 matrisomal proteins were exclusively identified by Hill's method (Figure 3D and Table 1). These 9 proteins include the collagens XI (COBA1) and XV (COFA1), the proteoglycan prolargin (PRELP), the ECM-affiliated protein annexin 6 (ANXA6) and four ECM regulators: three forms of α-1-antitrypsin (A1AT1, A1AT2 and A1AT4) and serpin H (SERPH). PRELP has previously been identified by O'Brien et al. [34] in rat mammary glands, whereas COBA1, COFA1, ANXA6, A1AT and SERPH have been detected in mouse mammary fat pad xenograft models of breast carcinoma [18].

Proteins of the core matrisome were the dominant matrisomal proteins identified in the enriched mammary gland samples (Figure 3E). For example, 15 collagens were identified by the EMA protocol, which is consistent with the previously reported study by O'Brien et al. [34], where 19 collagens were identified in rat mammary gland using the same enrichment method. In the glycoprotein class of core matrisome proteins, eight different laminin chains were identified by O'Brien et al. [34] compared with four chains in our analysis. Overall, 60 matrisomal proteins were identified in the study by O'Brien et al. versus 41 identified in our dataset. However, it should be noted that O'Brien et al. performed extensive fractionation of the sample into 17 fractions by SDS–PAGE prior to LC–MS/MS analysis, whereas our study has no fractionation steps after matrisome enrichment. This extensive fractionation may account for the higher number of matrisomal proteins identified in the O'Brien et al. study.

Unexpectedly, the four enrichment methods did not dramatically increase the number of identified core matrisomal proteins when compared with the non-enriched samples (Figure 3E). A total of 30 core matrisome components were identified in the non-enriched sample, whereas 29 proteins were identified after Triton X-100 decellularisation, 23 after SDS decellularisation, 34 after extraction by the EMA protocol and 30 after extraction by the EMB protocol. Increasing the liquid chromatography gradient from 88 min to 4 h increased the number of identified core matrisomal proteins in the non-enriched sample to 39. Of the four enrichment strategies, the EMA protocol yielded the highest number of seven matrisome-associated proteins. However, similar to the heart, matrisome-associated proteins were poorly enriched by the four methods compared with non-enriched samples in the mammary gland (Figure 3E).

### Lung

The lung is a complex organ composed of airways and alveoli intertwined with veins. The lung ECM is primarily composed of collagen and elastin fibres. Because of the requirements for very high flexibility, elastin is placed among individual alveoli and capillaries where it contributes to lung elasticity during inhalation and plays a major role in the intrinsic recoil of the lung [35]. The more rigid ECM molecules of collagen I, II and III are found in the bronchus, bronchioles and veins, whereas collagen IV and V are present in the basement membrane of capillaries and alveoli [35]. Lung-specific ECM components include the surfactants which consist of lipoproteins that are present in the alveoli where they decrease the air–liquid tension and thus protect alveoli from collapse [36].

**Figure 4.**
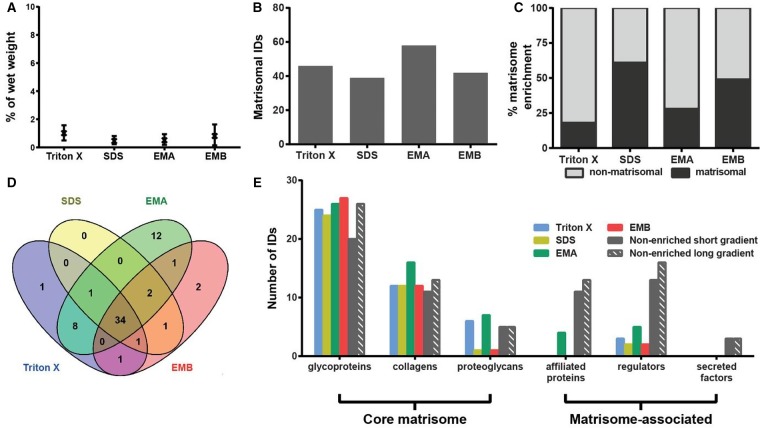
Comparison of methods for enrichment of the lung matrisome. (**A**) Percentage protein extraction yield after enrichment by individual methods. Results from three biological replicates are shown as mean and range (*n* = 3). (**B**) Number of matrisomal proteins identified (IDs) after enrichment. (**C**) Proportional representation of identified matrisomal and non-matrisomal proteins after enrichment. (**D**) Venn diagram depicting the overlap of identified matrisomal proteins across the individual enrichment methods. (**E**) Distribution of proteins identified (IDs) in matrisomal classes for enriched and non-enriched samples. Only proteins detected in at least two biological replicates were considered.

The average protein extraction yield in murine lung after matrisome enrichment was 0.5–1% wet weight, with no statistically significant differences between the four methods (Figure 4A). Enrichment by the EMA protocol identified the largest number of 58 matrisomal proteins (Figure 4B), while the highest level of matrisome enrichment of 61% was achieved after SDS decellularisation (Figure 4C). The number of proteins that were identified using the EMB protocol in our study (between 37 and 70 proteins in three independent biological replicates) is comparable with the published study by Naba et al. [9], where 55 matrisomal proteins were identified in murine lungs in one biological replicate in the absence of fractionation by off-gel electrophoresis. Collectively, 42 matrisomal proteins were identified in at least 2 of 3 biological replicates using the EMB protocol in our study.

Comparative analysis revealed 34 matrisomal proteins that were commonly identified by all four enrichment methods (Figure 4D). Twelve proteins were identified exclusively after extraction by the EMA protocol. Annexin 2 (ANXA2), A1AT, Galectin-3 (LEG3) and pulmonary surfactant-associated proteins A and B (SFTPA, PSPB) were among the proteins identified exclusively using this method (Table 1). These proteins have previously been identified in published murine lung matrisome studies [9,13]. Additionally, the ECM regulator plasminogen (PLMN) was detected exclusively in Triton X-100 decellularisation and the glycoprotein latent-TGF-β-binding protein 4 (LTBP4) was only found in the EMB protocol. Similar to the heart and mammary gland, collagens and glycoproteins were the most prominent classes of matrisomal proteins that were recovered (Figure 4E). Interestingly, Triton X-100 decellularisation and EMA extraction were superior in the enrichment of proteoglycans in the lung (Figure 4E). Consistent with our analyses in the other organs, the four matrisome enrichment methods led to significant losses in matrisome-associated proteins compared with the non-enriched samples (Figure 4E).

### Liver

**Figure 5.**
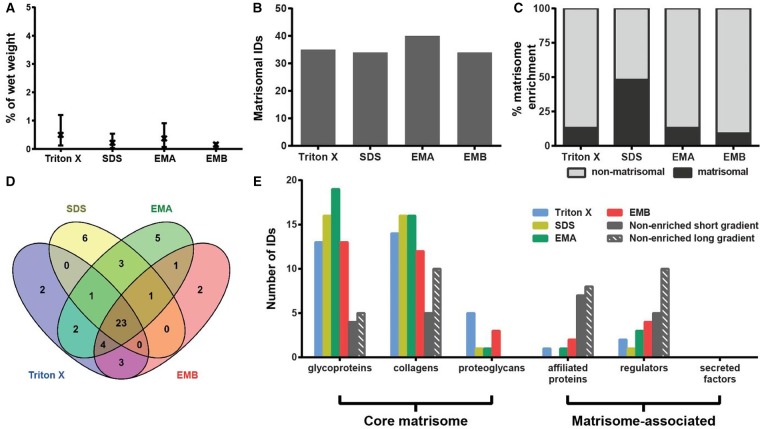
Comparison of methods for enrichment of the liver matrisome. (**A**) Percentage protein extraction yield after enrichment by individual methods. Results from three biological replicates are shown as mean and range (*n* = 3). (**B**) Number of matrisomal proteins identified (IDs) after enrichment. (**C**) Proportional representation of identified matrisomal and non-matrisomal proteins after enrichment. (**D**) Venn diagram depicting the overlap of identified matrisomal proteins across the individual enrichment methods. (**E**) Distribution of proteins identified (IDs) in matrisomal classes for enriched and non-enriched samples. Only proteins detected in at least two biological replicates were considered.

The ECM of the liver comprises <3% of the normal liver and is limited to the Glisson's capsule, portal tracts and central veins [37]. Unlike other organs, there is no physical basement membrane surrounding hepatocytes, although the individual proteins of the basement membrane are present [37,38]. Instead, hepatocytes are surrounded directly by matrix consisting of FINC, collagens and basement membrane proteins, which facilitate rapid diffusion between the blood and cells [39]. The low ECM levels in the liver may be a possible explanation for the low protein extraction yields of between 0.17 and 0.5% average wet weight, with no statistically significant differences between the four methods (Figure 5A). The highest number of 40 matrisomal proteins was identified by the EMA method (Figure 5B) and the highest percentage of matrisome enrichment was obtained by SDS decellularisation, where 48% of total proteins were assigned as matrisomal (Figure 5C). A total of 23 proteins were common across the four employed methods (Figure 5D and Table 1). Many proteins were uniquely identified by specific methods. For instance, SDS decellularisation identified six unique matrisomal proteins including laminins β3 (LAMB3) and γ2 (LAMC2), LTBP4 and three types of collagens (CO4A1, CO7A1 and CODA1). The EMA protocol found five unique proteins such as laminins α4 (LAMA4) and β1 (LAMB1), collagens XVI (COGA1) and XVIII (COIA1) and α-1-antitrypsin (A1AT2). Triton X-100 decellularisation identified two proteoglycans lumican (LUM) and decorin (PGS2), while the EMB protocol identified two matrisome-associated proteins ERGIC-53 (LMAN1) and serine protease inhibitor A3K (SPA3K).

The benefit of matrisome enrichment in the liver is pronounced compared with non-enriched samples, where a short gradient identified less than half of the proteins in the core matrisome recovered by the matrisome enrichment methods (Figure 5E). For example, no proteoglycans were identified in non-enriched samples, compared with three proteoglycans — PGBM, byglican (PGS1), PRELP — identified after the EMB protocol extraction and five proteoglycans (PGBM, LUM, PGS1, PGS2 and PRELP) identified after Triton X-100 decellularisation (Table 1). Importantly, our findings demonstrate that enrichment is necessary for comprehensive analysis of the liver matrisome, which may be the result of the low ECM content in this organ.

### Comparative assessment across organs

**Figure 6.**
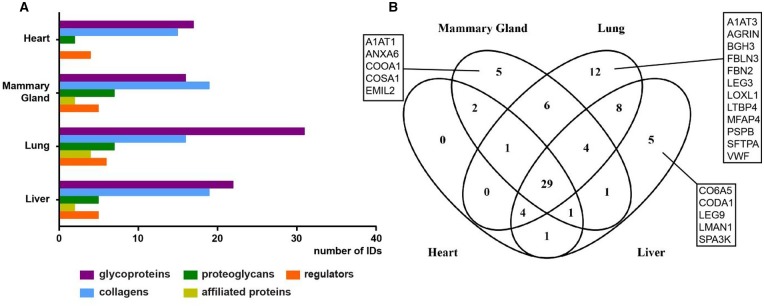
Comparative assessment of matrisomal proteins across organs. (**A**) Distribution of identified proteins in matrisomal classes enriched by all methods across the four organs. Proteins identified in two or more biological replicates were considered. (**B**) Venn diagram depicting the overlap of identified matrisomal proteins across the individual organs.

Across the four organs examined, the highest number of matrisomal proteins was detected in the lung tissue, where 64 proteins were identified, 12 of which were unique to the lung (Figure 6A,B). In particular, glycoproteins were found in high abundance in lung compared with other organs (Figure 6A), with the following lung-specific glycoproteins identified in our study: agrin (AGRN), fibrillin-2 (FBN2), fibulin-3 (FBLN3), TGF-β-induced protein ig-h3 (BGH3), LTBP4 and von Willebrand factor (VWF; Figure 6B). In contrast, matrisomal enrichment of the heart showed the lowest number of 38 matrisomal proteins identified with only few proteoglycans and no affiliated proteins detected (Figure 6A). Furthermore, unlike the other three organs, no heart-specific proteins were identified in our study (Figure 6B). In addition, glycoprotein emilin-2 (EMIL2), collagens XXIV (COOA1) and XXVIII (COSA1), A1AT1 and ANXA6 were uniquely identified in mammary glands, while α5 chain of type VI collagen (CO6A5) and α1 chain of type XIV collagen (CODA1), affiliated proteins galectin-9 (LEG9) and LMAN and regulator SPA3K were unique to liver tissue.

Our analysis finds 29 matrisomal proteins that are common across all four organs (Figure 6B). These common matrisome components comprise primarily proteins of the collagen and glycoprotein classes (Table 1). The identification of common matrisome proteins in the present study will contribute to the efforts by several groups to comprehensively define the ‘ECM atlas’ across multiple tissue types [10]. It should be noted that our study is qualitative in nature and does not provide any quantitative information of the relative levels of the 29 matrisomal proteins in each of the four organs examined. This list of common matrisomal components will be useful for the development of selected reaction monitoring (SRM) approaches for quantitative profiling of the matrisome in multiple tissue types, where the identity of the proteins and peptides of interest are required *a priori* for assay development [40]. A recent study describes the development of an SRM method based on QconCat technology, which utilises 83 stable isotope-labelled peptides representing 48 different ECM proteins to measure decellularised human hearts [14]. Of our 29 common matrisomal proteins, 60% are found in the QconCat library, indicating that this quantitative SRM method can be readily extended to quantitative matrisomal measurements in lung, mammary gland and liver.

### Comparative assessment across methods

**Figure 7.**
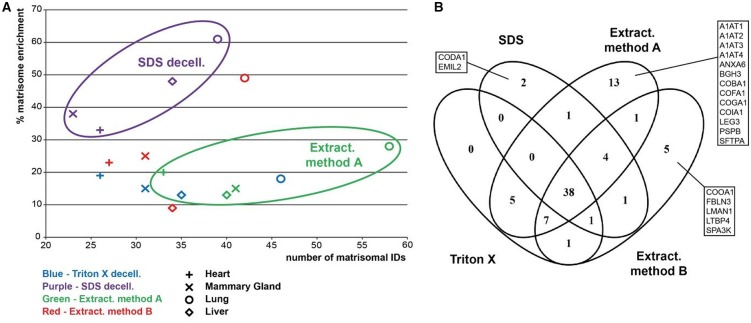
Comparative assessment of matrisomal proteins across enrichment methods. (**A**) A scatter plot of the number of identified matrisomal proteins and enrichment capability. Clustering of samples extracted by the EMA method and after SDS decellularisation is highlighted. The percentage matrisome enrichment is defined as the percentage of identified proteins which are catalogued in the Matrisome Project Database. Only proteins detected in at least two biological replicates were considered. (**B**) Venn diagram depicting the overlap of identified matrisomal proteins across the individual enrichment methods.

To evaluate the performance of individual methods across the four organs, a scatter plot displaying percentage matrisome enrichment versus the number of identified unique matrisomal proteins was generated (Figure 7A). Our study finds that while SDS decellularisation provides the highest percentage matrisome enrichment across all four organs, the number of identified matrisomal proteins is consistently the lowest. This result may be due to the negative charge of SDS, which causes protein denaturation and tissue disintegration. While this property of SDS facilitates the effective removal of the cellular proteins during washing steps, it also leads to losses in matrisomal proteins, leading to our observed high percentage matrisome enrichment accompanied by low numbers of matrisome protein identifications. Consistent with this idea, the loss of matrisomal glycosaminoglycans and reduced collagen integrity after SDS decellularisation have been described in aortic valves, annulus fibrosus and rat tail [12,25,41,42].

The EMA extraction protocol identified the highest number of matrisomal proteins in all analysed tissues, despite having similar percentage matrisomal enrichment as Triton X-100 decellularisation and the EMB protocol (Figure 7A,B). The capability of the EMA protocol to identify more matrisomal proteins is also reflected by the number of proteins identified exclusively by this method, particularly in lung and mammary gland (Figure 7B and Table 1). For example, A1AT2 was identified exclusively by the EMA protocol in all analysed tissues. In addition, SFTPA, PSPB and ANXA6 were identified only in samples after extraction with this method. The percentage matrisome enrichment and the number of matrisomal proteins identified by Triton X-100 decellularisation and the EMB protocol were very similar across all types of tissues (Figure 7A). The only exception is lung tissue, where the EMB extraction protocol provided superior matrisomal enrichment compared with Triton X-100 decellularisation.

There is a significant overlap of identified matrisomal proteins between methods across all analysed tissues, with 38 of 79 matrisomal proteins commonly identified by all four methods (Figure 7B). These proteins include important core matrisomal glycoproteins [such as fibrillin 1 (FBN1), fibrinogens (FIBA, FIBB and FIBG), FINC and laminins] and collagens (Table 1). Furthermore, in agreement with previous studies showing that Triton X-100 improves the recovery of proteoglycans [43], our experiments also demonstrate that Triton X-100 decellularisation provided superior enrichment of the proteoglycan class of matrisomal proteins across all four organs (Figures 2E, 3E, 4E and 5E).

In terms of the number of unique matrisome proteins identified, our study shows that lung and mammary gland do not appear to benefit from matrisome enrichment as direct LC–MS/MS analysis of the non-enriched samples leads to the identification of similar or higher numbers of core matrisome components (Figures 3E and 4E). One potential reason for this unexpected finding is that these two tissue types have higher levels of overall ECM content compared with heart and liver. Taking collagen content as an indicator for overall ECM content in an organ, the content of collagen in rat lung is 11.3% of dry fat-free weight, whereas rat ventricle contains 2.96% and rat liver 0.64% [44]. In rat mammary gland, collagen forms 9.9% of wet fat-free weight, whereas the content of non-collagenous protein is 10 times lower [45]. The high (lung and mammary gland) and low (heart and liver) collagen content — and thus an approximation of ECM content in the tissue — is in good agreement with the requirement for matrisome enrichment in our study. For the latter two organs, direct LC–MS/MS analysis of unenriched samples is sufficient to identify the abundant core matrisomal proteins.

It has been suggested that matrisome enrichment is required to increase the depth of protein sequence coverage due to the large number of ECM proteins displaying a wide dynamic range [11]. We sought to determine if non-enriched samples have reduced sequence coverage by taking a selection of four proteins from distinct matrisomal classes and plotting the percentage protein sequence coverage across all methods and organs (Supplementary Figure S1). We find that there is no clear relationship between sequence coverage and the type of enrichment. While the non-enriched samples show reduced protein sequence coverage across all organs in some proteins (FINC and PGBM), this is not always the case. For instance, in the case of CO1A1 (Supplementary Figure S1C), the non-enriched samples display comparable sequence coverage to the other four enrichment methods in all organs examined except the heart. In another example of TGM2 (Supplementary Figure S1D), the non-enriched samples have higher or similar sequence coverage compared with EMA in the lung and liver but lower sequence coverage in the heart and mammary gland. These data demonstrate that percentage sequence coverage is not always reduced in non-enriched samples and is likely dependent on the type of organ, method of choice and protein of interest.

Our comparative analysis also revealed that a large proportion of matrisome-associated proteins are lost during enrichment using any of the four methods. These results are perhaps expected given that the majority of proteins in the matrisome-associated class are soluble and readily lost during enrichment. In the deep proteomic study of mouse lung ECM by Schiller et al. [16], only 53 of a total 264 matrisome-associated proteins were found to be enriched in the insoluble fraction after quantitative detergent solubility profiling. Similarly, in a study of the human ventricle ECM performed by Barallobre-Barreiro et al. [46], a higher abundance of matrisome-associated proteins such as S100 proteins or ECM peptidases was found in the soluble fraction. Our data highlight that in-depth proteomic analysis of non-enriched samples is currently the best available tool for the analysis of this class of proteins; however, further development of new enrichment strategies to better isolate matrisome-associated proteins with improved sequence coverage is necessary.

## Conclusion

In summary, we have performed a comparative proteomic analysis of four matrisome enrichment strategies in four different organs. Our data emphasize that the choice of enrichment strategy is dependent on tissue type, matrisome class of interest and desired matrisome enrichment purity. Based on our results, SDS decellularisation is a good option for attaining high purity of matrisome-enriched samples, while the EMA extraction protocol can be universally used to achieve high levels of matrisome protein identification. Triton X-100 decellularisation provides good enrichment of proteoglycans, whereas in-depth proteomic analysis of non-enriched samples is necessary to identify matrisome-associated proteins. We further show that, in some organs such as mammary glands and lungs, matrisome enrichment is not superior to proteomic analysis of non-enriched samples based on the number of matrisomal proteins identified and suggest that this may be due to the overall higher content of ECM in these tissues. We anticipate that future work comparing matrisome enrichment versus non-enriched samples in additional tissue types of differing ECM content will provide more clarity of the necessity for prior sample enrichment in the proteomic characterisation of the matrisome.

## Abbreviations

A1AT: α-1-antitrypsin
ABC: ammonium bicarbonate
ACN: acetonitrile
ANXA2: Annexin 2
BCA: bicinchoninic acid
BGH3: β-induced protein ig-h3
CHAPS: 3-[(3-cholamidopropyl)dimethylammonio]-1-propanesulfonate
ECM: extracellular matrix
EDTA: ethylenediamine tetraacetic acid
EMA: Extraction method A
EMB: Extraction method B
FA: formic acid
FBLN: fibulins
FBN1: fibrillin 1
FBN2: fibrillin-2
FBLN3: fibulin-3
FDR: false discovery rate
FINC: fibronectin
KIU: Kallikrein inhibitor unit
LC–MS/MS: liquid chromatography–tandem mass spectrometry
LEG3: Galectin-3
LTBP4: latent-TGF-β-binding protein 4
LUM: lumican
MS/MS: tandem mass spectrometry
PBS: polybuffered saline
PGBM: perlecan
PGS2: decorin
PLMN: plasminogen
SDS: sodium dodecyl sulphate
SERPH: serpin H
SFTPA and PSPB: pulmonary surfactant-associated proteins A and B
SRM: selected reaction monitoring
TFA: trifluoroacetic acid
TGF-β: transforming growth factor β
VWF: von Willebrand factor.
